# Enhanced Lethal Effects of Combined P-tert-Butylcatechol and L-Lysine on *Microcystis aeruginosa*

**DOI:** 10.3390/biology14060655

**Published:** 2025-06-05

**Authors:** Heyun Jiao, Gangwei Jiao, Ruitong Jiang, Yifei Shen, Peimin He, Liu Shao

**Affiliations:** 1 College of Oceanography and Ecological Science, Shanghai Ocean University, Shanghai 201306, China; j15039800350@163.com (H.J.); gangweijiao2021@163.com (G.J.); asharjj@outlook.com (R.J.); shenyifei317@163.com (Y.S.); 2 Marine Scientific Research Institute, Shanghai Ocean University, Shanghai 201306, China

**Keywords:** allelochemicals, algae inhibition, microcystins, photosynthetic system, oxidative stress response

## Abstract

Allelochemicals are recognized as promising algaecides due to their environmental safety. Para-tert-butylcatechol (TBC) and L-lysine exhibit significant potential in suppressing harmful algal blooms (HABs); however, their combined effects and algae inhibition mechanisms remain unelucidated. Therefore, this study systematically investigated the growth inhibition of *Microcystis aeruginosa* by TBC and L-lysine individually and in combination, while simultaneously examining their combined effects on algal growth, cell membrane integrity, photosynthetic activity, antioxidant responses, and microcystin production. The results revealed a significant interactive effect between TBC (0.04 mg/L) and L-lysine (1 mg/L), achieving over 90% growth inhibition within 96 h. The combined treatment significantly inhibited *M. aeruginosa* growth through impaired photosynthetic efficiency and elevated oxidative stress. Compared to the control group, the treatment group exhibited a continuous decline in chlorophyll-a content, phycobiliprotein levels, *F_v_*/*F_m_*, YII, α, and rETR_max_, while phosphoenolpyruvate carboxylase (PEPC) activity decreased by 96.48% by day 8. And antioxidant enzymes, including superoxide dismutase (SOD) and reduced glutathione (GSH), showed a progressive increase in activity. In addition, the structure and integrity of the cell membrane of *M. aeruginosa* were damaged after treatment, and the conductivity of the treatment groups increased continuously from 2.32 to 4.63 μs/cm. In addition, under combined treatment, intra- and extracellular microcystin levels initially increased (peaking at day 2) but sharply declined thereafter, becoming significantly lower than controls by day 8. These findings highlight the potential of combining TBC and L-lysine as an eco-friendly and cost-effective strategy for mitigating *M. aeruginosa*-dominated harmful algal blooms.

## Simple Summary

Harmful algal blooms caused by *Microcystis aeruginosa* threaten aquatic ecosystems and human health. This study explores the combined effects of two natural allelochemicals, P-tert-butylcatechol (TBC) and L-lysine, in controlling *M. aeruginosa*. When combined at low concentrations (0.04 mg/L TBC + 1 mg/L L-lysine), they achieved over 90% growth inhibition within 96 h. The treatment damaged algal cell membranes, reduced photosynthetic efficiency, and triggered oxidative stress. Furthermore, intracellular and extracellular microcystin levels decreased significantly compared to untreated algae, minimizing ecological risks. These findings highlight the potential of the combination of TBC and L-lysine as a safe and cost-effective strategy for mitigating harmful algal blooms.

## 1. Introduction

In recent years, excessive nutrient inputs into aquatic ecosystems, driven by global climate change and anthropogenic activities, have led to progressively intensified eutrophication [1,2]. Eutrophication promotes the rapid growth of algae and the formation of harmful algal blooms (HABs). HABs can rapidly reduce water transparency and lower the dissolved oxygen level, leading to wildlife mortality. This imbalance in the food chain ultimately leads to the collapse of the ecosystem [3].

*Microcystis aeruginosa*, one of the most dominant and widely distributed cyanobacteria during bloom outbreaks, is also the primary producer of common cyanotoxins [4]. The microcystins released by *M. aeruginosa* pose a serious threat to aquatic life and human health, degrade water quality, impact the fishery and aquaculture industries, increase water treatment costs, and hinder social and economic development [5]. Consequently, developing effective strategies to control *M. aeruginosa* blooms in eutrophic waters has become a critical research priority, necessitating targeted approaches to mitigate cyanobacterial-dominated HABs in global aquatic systems.

Physical, chemical, and biological methods have been extensively studied and applied to inhibit HABs [6]. Compared to physical and chemical methods, biological methods offer advantages such as ease of implementation, effective results, and minimal environmental impact [7]. Among these, harnessing allelopathy to suppress HABs presents a promising avenue for developing efficient and safe anti-algal strategies [8]. Recently, various allelochemicals with inhibitory effects on algae have been extracted and isolated from different plants, including organic acids, simple phenols, polyphenols, amines, lipids, and other organic compounds [9]. To further enhance the algal inhibition efficiency of allelochemicals, many researchers have started exploring the combined application of multiple allelochemicals [10]. This approach can synergistically leverage the advantages of different components, allowing for more efficient, safe, and cost-effective management of HABs [11,12]. The combined effects of allelochemicals refer to the biological responses induced by two or more allelopathic substances, which can be categorized into four distinct interaction types: synergistic (where the combined effect is greater than the sum of individual effects), additive (equal to the sum of individual effects), antagonistic (less than the sum of individual effects), and independent action (where compounds act through separate mechanisms without interaction) [13].

L-lysine, an essential nitrogenous amino acid for mammals, shows selective algicidal activity against toxic cyanobacteria like *Microcystis* sp. [14,15]. P-tert-Butylcatechol (TBC) is a phenolic secondary metabolite commonly found in plant tissues and has also been proven to inhibit the growth of *M. aeruginosa* [16]. Moreover, both allelochemicals exhibit high chemical stability [17,18]. However, the algicidal effects of their combined action remain unclear. In this study, we systematically investigated the inhibitory effects of TBC and L-lysine, both individually and in combination, on *M. aeruginosa*, along with the underlying mechanisms of their combined algicidal action. The specific objectives of this study are as follows: (1) the inhibitory effects of TBC and L-lysine, both individually and in combination, on *M. aeruginosa* were investigated; (2) the influence of combined algaecide on growth and integrity of cell membrane was detected; (3) the further determination of the impact on photosynthesis and the antioxidant system provided new insights into the mechanism of action of combined algaecide; and (4) the level of intracellular and extracellular microcystins in *M. aeruginosa* exposed to combined algaecide was analyzed. These findings provide novel insights in addressing eutrophication issues in water bodies, while also offering scientific basis and theoretical support for the prevention and control of *M. aeruginosa* blooms.

## 2. Materials and Methods

### 2.1. Algae Culture Conditions

*Microcystis aeruginosa* (FACHB-905) was purchased from the Freshwater Algae Culture Collection at the Institute of Hydrobiology (Wuhan, China). Algal cells were maintained under axenic conditions throughout the experiment. Algal cells were grown in 250 mL Erlenmeyer flasks containing sterilized BG11 medium under a 12:12 light/dark cycle (6000 lux, 25 ± 1 °C). Flasks were manually shaken three times daily to ensure uniform mixing.

### 2.2. Experimental Design

#### 2.2.1. Individual and Combined Algal Inhibition Effects of TBC and L-Lysine

L-lysine (purity ≥ 98%) was purchased from Aladdin Biochemical Technology Co., Ltd. (Shanghai, China), and TBC (purity ≥ 98%) was purchased from Macklin Biochemical Technology Co., Ltd. (Shanghai, China). To evaluate the dosage effects of TBC and L-lysine on *M. aeruginosa*, concentration gradients were established based on preliminary experiments: 0.01, 0.02, 0.03, 0.04, and 0.05 mg/L for TBC, and 1, 2, 4, 8, and 16 mg/L for L-lysine. Using pure water as the solvent, working solutions of L-lysine were prepared at concentrations of 1, 2, 4, 8, and 16 mg/mL, and working solutions of TBC were prepared at concentrations of 0.01, 0.02, 0.03, 0.04, and 0.05 mg/mL. For the treatment groups, 0.1 mL of each allelochemical working solution at different concentrations was added to 100 mL cultures of *M. aeruginosa* (initial cell density: 2 × 10^6^ cells/mL) in the logarithmic growth phase; for the control group, 0.1 mL of pure water was added instead.

The optimal combined ratio between TBC and L-lysine was determined through a two-phase optimization approach based on preliminary concentration gradient results. Phase I tested TBC (0.03 mg/L, EC_50_ at 96 h) combined with L-lysine (0, 1, 3 mg/L), identifying 0.03 mg/L TBC + 1 mg/L L-lysine as optimal. Phase II further optimized the ratio by testing 0.03, 0.04, and 0.05 mg/L TBC while maintaining the optimal L-lysine concentration of 1 mg/L. Blank control groups were established at each experimental phase. The initial algal density in every flask was 2.00 × 10^6^ cells/mL. All experiments were conducted under standardized algal culture conditions with three biological replicates per treatment group.

#### 2.2.2. Research on the Combined Effect of Algal Inhibition Mechanism

Through the experiments explained in Section 2.2.1, the optimal combined concentration of TBC and L-lysine for algal inhibition was selected to investigate their joint algicidal mechanism. Treatment groups were given 0.04 mg/L TBC + 1 mg/L L-lysine; blank control groups were established in parallel. Samples were taken on 0, 2, 4, 6, and 8 days for the mechanism study. The initial algal density in every flask was 2.00 × 10^6^ cells/mL. All the controls and the treatments are replicated 3 times and shaken 3 times by hand each day during the incubation.

### 2.3. Determination of Algal Cell Density

Cell density was measured using a hemocytometer under a light microscope (Nikon Corporation, Tokyo, Japan). Samples were collected every 2 days, and the inhibition rate (IR) was calculated as follows:

(1) $$\mathrm{IR}=\frac{C_{0}-C_{t}}{C_{0}}\times 100\%$$

where IR is the growth inhibition rate (%), *C*_0_ is the algae density of the control group, and *C_t_* is the algae density of the treatment group.

### 2.4. Determination of Chlorophyll-a Content and Chlorophyll Fluorescence

The chlorophyll-a (Chl-a) content was calculated spectrophotometrically. Chl-a content of *M. aeruginosa* was extracted by 90% acetone at 4 °C for 24 h under dark conditions according to Kong [19]. The formula for calculating Chl-a is as follows:

(2) $$\mathrm{Chl}-a\left(\mathrm{mg}/m^{3}\right)=\frac{\left[11.64\times \left(OD_{663}-OD_{750}\right)-2.16\times \left(OD_{645}-OD_{750}\right)+0.1\times \left(OD_{630}-OD_{750}\right)\right]\times V_{1}}{V\times \delta }$$

where V is the suction filtration volume (L); V1 is the volume of the extraction solution after the volume is fixed (mL); δ is the cuvette path length (cm); OD is the absorbance.

Chlorophyll fluorescence transients were measured using a pulse-amplitude-modulated (PAM) fluorescence monitoring system (Phyto-PAM, Walz, Nuremberg, Germany) after 0, 2, 4, 6, and 8 days of treatment. Algal samples were transferred into 3 mL centrifuge tubes and subjected to 15 min dark adaptation before measurements. Based on chlorophyll fluorescence transients, the relative electron transport rate (rETR) and *F_v_*/*F_m_* were obtained directly from the relative value of the plateau phase in the rapid light curve [20]. The other photosynthetic activity parameters (YII and α) were also tested every 48 h. YII reflects actual PSII photochemical efficiency under illumination; α quantifies algal solar energy utilization efficiency.

### 2.5. Determination of Cell Survival Status

Fluorescein diacetate (FDA, purity ≥ 97%) was purchased from Macklin Biochemical Technology Co., Ltd. (Shanghai, China), and propidium iodide (PI, 1 mg/L) was purchased from Beijing Solarbio Science & Technology Co., Ltd. (Beijing, China). The FDA was utilized to assess algal cell esterase activity, while PI served to evaluate cell membrane integrity. FDA and PI were added to the samples for staining. Cell viability was analyzed via flow cytometry (BD Biosciences, San Jose, CA, USA) with 488 nm laser excitation. Fluorescence emission was detected in FL1 (530/30 nm bandpass) for SYBR Green-stained viable cells and FL3 (>670 nm longpass) for PI-positive nonviable cells with membrane damage.

### 2.6. Determination of Soluble Protein Contents and Phycobiliprotein

Soluble protein content in *M. aeruginosa* was quantified via Coomassie Brilliant Blue G250 assay [21]. A protein standard curve was established to quantify the soluble protein content in the algal cells. The measured OD_595_ values of the unknown samples were applied to this calibration curve to ascertain the soluble protein contents.

The contents of phycocyanin (PC), allophycocyanin (APC), and phycoerythrin (PE) in algal cells were determined by the low-temperature freeze–thaw method. Algae cultures were sampled from each flask and centrifuged at 8000 r/min for 10 min at 4 °C. The algal sediments were resuspended with 5 mL phosphate-buffered saline (PBS, 0.05 mol/L, pH = 7.0). Following storage at −80 °C for 8 h in an ultra-low-temperature freezer, samples underwent sequential processing: dissolution under dark conditions at ambient temperature, followed by three freeze–thaw cycles, and centrifugation at 12,000 r/min for 10 min at 4 °C, with the supernatant liquid subsequently collected. The contents of phycobiliproteins, including PC, APC, and PE, were measured at 565, 620, and 650 nm, respectively. The calculation formulas are as follows:

(3) $$C_{\mathrm{PC}}=\left(OD_{620}-0.7\times OD_{650}\right)/7.38$$

(4) $$C_{\mathrm{APC}}=\left(OD_{650}-0.19\times OD_{620}\right)/5.65$$

(5) $$C_{\mathrm{PE}}=(OD_{565}-2.8\times \mathrm{PC}-1.34\times \mathrm{APC})/12.70$$

where CPC, CAPC, and CPE are the PC, APC, and PE concentrations, respectively; OD is the absorbance.

### 2.7. Determination of Membrane Permeability

The method for determining protein (OD_280_), nucleic acid (OD_260_), and conductivity (EC) in algal fluid was as follows [22]: 20 mL of algae solution was taken and placed in a high-speed centrifuge at 6000 r/min for 10 min to obtain the supernatant. Supernatant aliquots were analyzed spectrophotometrically at 260 nm (nucleic acids) and 280 nm (proteins), with residual fractions used for EC measurement. The OD values from the experimental and control groups were employed to evaluate the release level of nucleotides and protein.

### 2.8. Determination of Enzyme Activity and Microcystins (MCs)

To investigate the effects of combined TBC and L-lysine on the oxidative stress, photosynthetic system, and MCs of *M. aeruginosa*, the activities of *M. aeruginosa*-related enzymes and the content of MCs were detected. A 50 mL sample of algae solution was placed in a sterilized centrifuge tube and centrifuged at 4 °C and 8000 r/min for 10 min, the supernatant was then utilized for extracellular MCs quantification. Pelleted cells underwent dual PBS washes (50 mmol/L, pH 7.0), followed by resuspension in 5 mL ice-cold PBS and sonication (10 min, 4 °C ice bath). After centrifugation at 4 °C and 8000 r/min for 10 min, the supernatant was taken as the crude enzyme extract. The crude enzyme extract was stored in a 4 °C freezer to determine the algal cells’ enzyme activity and intracellular MCs content.

Superoxide dismutase (SOD), glutathione (GSH), and phosphoenolpyruvate carboxylase (PEPC) activities were determined using the kit provided by Nanjing Jiancheng Biotechnology Research Institute Co., Ltd. (Nanjing, China). MCs were determined using a kit provided by Shanghai Enzyme-linked Biotechnology Co., Ltd. (Shanghai, China).

### 2.9. Statistical Analysis of Data

All experimental and control groups were set up in 3 parallel groups, and the experimental data were expressed as mean (X) ± standard deviation (SD). Microsoft Excel, Origin 8.0, and IBM SPSS Statistics 26.0 are used for data processing, statistical analysis, and graphing. Concentration–response curves derived from allelochemical gradient experiments were analyzed via probit regression to calculate EC_50_ values. For data being normally distributed and with homogeneous variance, statistical differences between the control and treated groups were analyzed using one-way analysis of variance (ANOVA) followed by a Tukey test. *p*-values of <0.05 and 0.01 indicate significant and highly significant differences, respectively.

## 3. Results

### 3.1. Effects of TBC and L-Lysine on M. aeruginosa Growth

**Figure 1:**
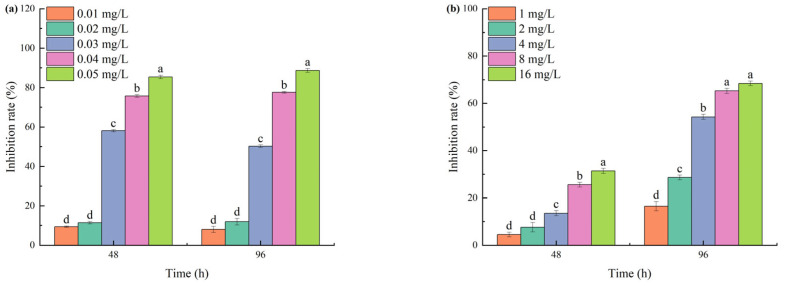
The inhibition of TBC and L-lysine on the growth of *M. aeruginosa*: (**a**) is TBC and (**b**) is L-lysine. Different letters indicate a significant difference at *p* < 0.05.

As the concentrations of TBC and L-lysine increased, and the culture duration was extended, the cell density of *M. aeruginosa* significantly decreased compared to the control group. At 0.02 mg/L TBC, growth inhibition reached only 11.92% after 96 h. When TBC concentrations were increased to 0.03, 0.04, and 0.05 mg/L, the 96 h inhibition rates reached 50.24%, 77.61%, and 88.65%, respectively (Figure 1a). TBC exhibited a 96 h EC50 value of 0.03 mg/L against *M. aeruginosa*. A measure of 1 mg/L L-lysine produced 15.06% inhibition after 96 h. When L-lysine concentration increased to 16 mg/L, the 96 h inhibition rate reached 68.45% (Figure 1b). L-lysine showed a higher 96 h EC50 (3 mg/L) compared to TBC.

### 3.2. Effects of Combined TBC and L-Lysine on M. aeruginosa Growth

**Figure 2:**
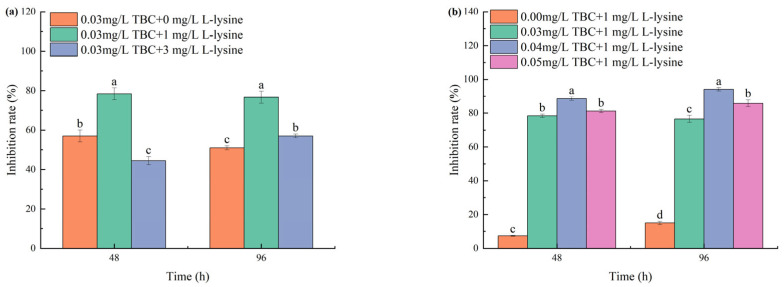
The inhibition of combined TBC and L-lysine on the growth of *M. aeruginosa*: (**a**) is Phase I and (**b**) is Phase II. Different letters indicate a significant difference at *p* < 0.05.

Compared to the control group, various ratios of combined TBC and L-lysine showed significant inhibitory effects on the growth of *M. aeruginosa*. In Phase I experiments (Figure 2a), the combination of 0.03 mg/L TBC with 1 mg/L L-lysine achieved 78.4% growth inhibition of *M. aeruginosa* at 48 h. By 96 h, the 0.03 + 1 mg/L combination maintained higher efficacy (76.67%) than 0.03 + 3 mg/L (57.03%). In Phase II (Figure 2b), the inhibitory effects of the 0.03 + 1, 0.04 + 1, and 0.05 + 1 mg/L TBC and L-lysine combinations showed a progressively increasing trend with prolonged exposure time, with 96 h growth inhibition rates reaching 76.67%, 94.14%, and 85.83%, respectively.

### 3.3. Identification of Viable Microcystis aeruginosa Cells by Flow Cytometry

**Figure 3:**
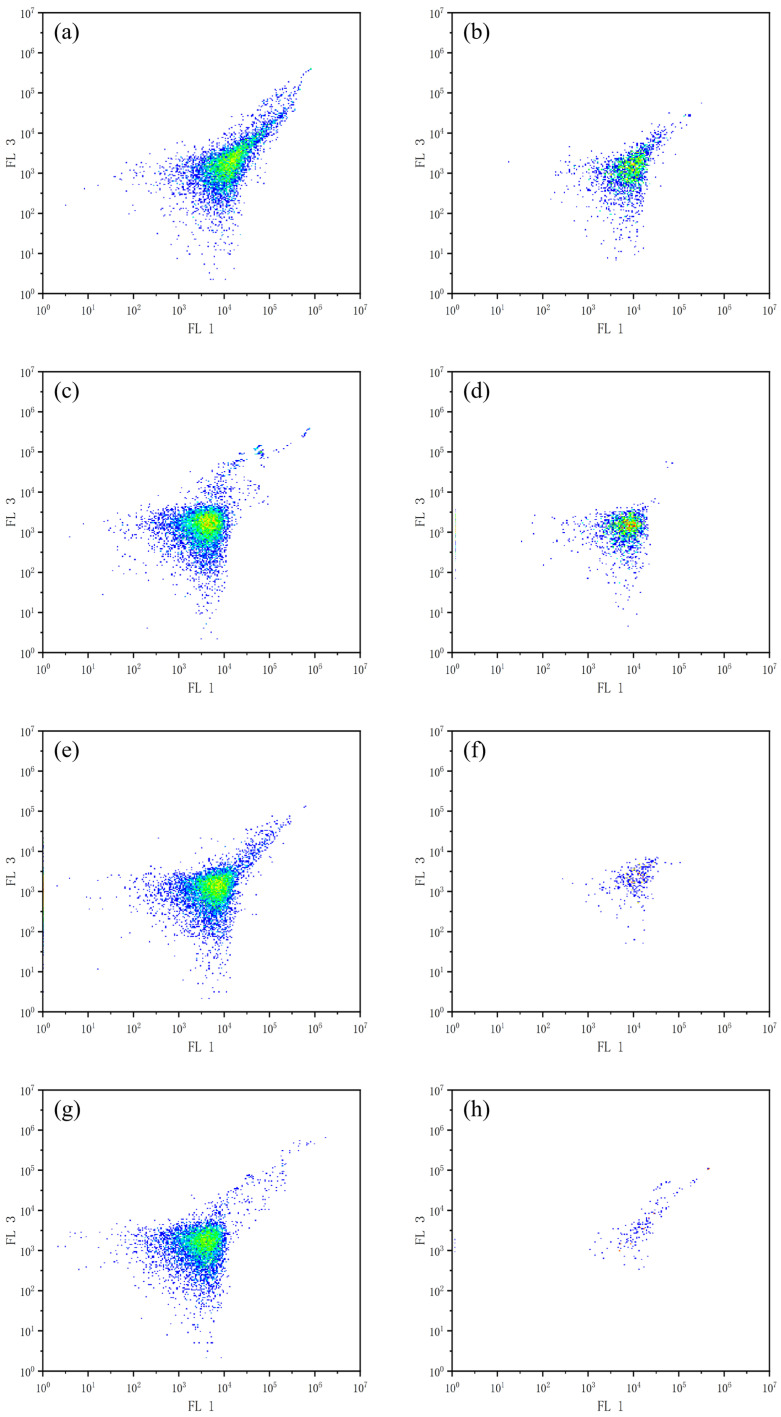
The effect of combined TBC and L-lysine on the survival cells of *M. aeruginosa*. (**a**,**c**,**e**,**g**) are control group, (**b**,**d**,**f**,**h**) are treatment group, a, and b are 2 d, c, and d are 4 d, e, and f are 6 d, g, and h are 8 d. The x-axis represents the FL 1 pathway and the y-axis represents the FL 3 pathway. Cellular density is represented by a chromatic scale transitioning from central red (high density) to peripheral blue (low density).)

Flow cytometric analysis using FDA/PI dual-staining was performed to evaluate the effects of combined allelochemicals on the cell viability of *M. aeruginosa*, as demonstrated in Figure 3. Analysis revealed significantly reduced viable cells in treated groups versus controls, with progressive depletion over time. When TBC and L-lysine were used in combination, the number of viable algal cells gradually decreased from day 2 to day 8. The experimental results indicate that the combination of TBC and L-lysine can significantly inhibit the growth of *M. aeruginosa* and may also disrupt cell membrane integrity.

### 3.4. Effects of Combined TBC and L-Lysine on the Photosynthetic System of M. aeruginosa

#### 3.4.1. Effects on Chlorophyll Fluorescence Transients

**Figure 4:**
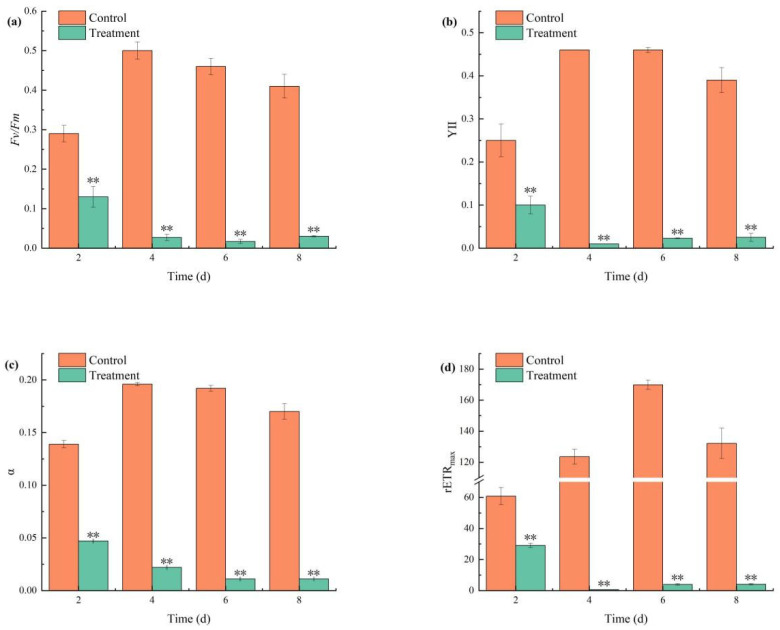
The effects of combined TBC and L-lysine on the parameters F_v_/F_m_ (**a**), YII (**b**), α (**c**), and rETR_max_ (**d**) deviated from the chlorophyll fluorescence parameters. Note: ** represent statistically significant differences with respect to the values of control cultures at *p* < 0.01 levels.

During the 2- to 4-day period of combined TBC and L-lysine treatment, all measured photosynthetic parameters (*F_v_*/*F_m_*, YII, α, and rETR_max_) exhibited significant decreasing trends. The *F_v_*/*F_m_* ratio in the treatment group fell below 0.05 starting on day 4 (Figure 4a), while the YII value decreased to 0.01 by day 4 and subsequently stabilized at 0.2 (Figure 4b). α showed progressive reductions of 66.19%, 88.77%, 94.27%, and 93.52% on days 2, 4, 6, and 8, respectively (Figure 4c). rETR_max_ plummeted by 96.93% by day 8, reflecting near-complete collapse of photosynthetic electron transport (Figure 4d).

#### 3.4.2. Effects on Chl-a and Soluble Protein

**Figure 5:**
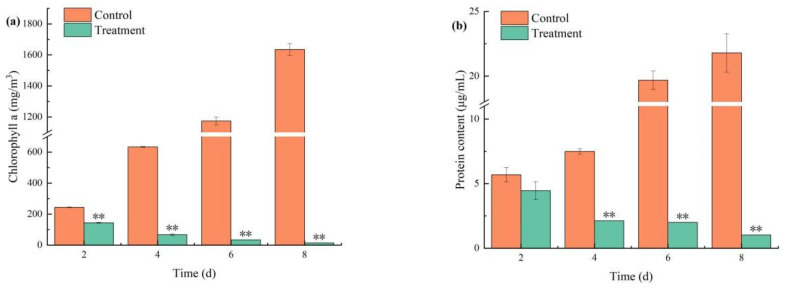
The effects of combined algaecide on the Chl-a (**a**) and protein (**b**) content of *M. aeruginosa*. Note: ** represent statistically significant differences with respect to the values of control cultures at *p* < 0.01 levels.

Combined treatment significantly reduced Chl-a content (*p* < 0.01; Figure 5a). It was observed that Chl-a levels decreased after 2 days of treatment with combined TBC and L-lysine. Compared to the control, combined TBC and L-lysine exhibited a highly significant inhibitory effect on Chl-a (*p* < 0.01). Chl-a declined continuously in the treatment group: 143.99, 67.21, 33.66, and 13.34 mg/m^3^ on days 2, 4, 6, and 8, respectively. Meanwhile, the inhibitory effect of combined TBC and L-lysine on algal Chl-a content strengthened over time, indicating a time-dependent effect. Soluble protein content, which serves as an indicator of biomass, can indicate the size and survival status of algal cells. The changes in soluble protein content in *M. aeruginosa* are consistent with that of Chl-a (Figure 5b), showing a continuous increasing trend in the control group. From day 4 to day 8, the soluble protein content in the treatment group significantly decreased by 71.66%, 89.84%, and 95.30%, respectively (*p* < 0.01).

#### 3.4.3. Effects on Phycobiliprotein Content

**Figure 6:**
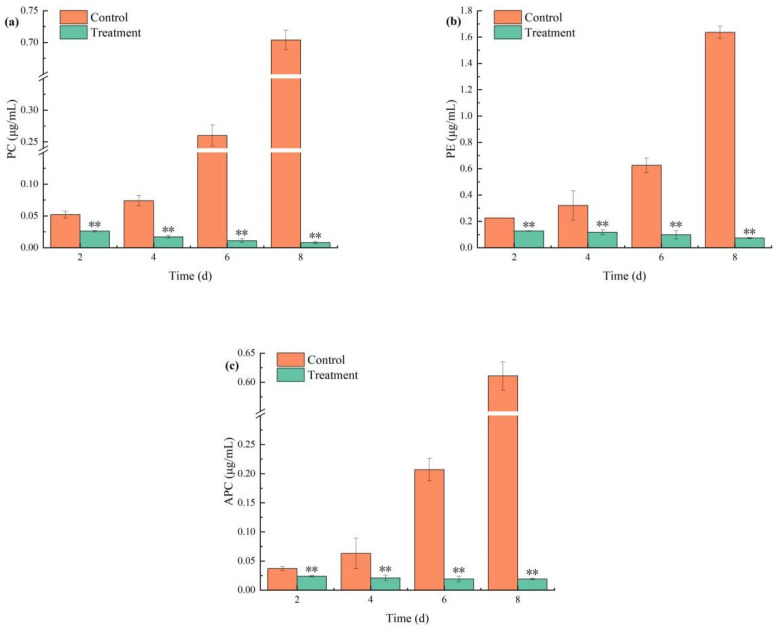
The effects of combined TBC and L-lysine on PC (**a**), PE (**b**), and APC (**c**) of *M. aeruginosa*. Note: ** represent statistically significant differences with respect to the values of control cultures at *p* < 0.01 levels.

The effects of combined TBC and L-lysine on the content of PC, APC, and PE in *M. aeruginosa* are illustrated in Figure 6. PC, APC, and PE concentrations increased gradually in controls but were significantly suppressed by combined treatment (Figure 6). On day 8, compared to the control group, the relative inhibition rates of PC, APC, and PE were 98.83%, 96.87%, and 95.55%, respectively.

#### 3.4.4. Effects on PEPC Activity

**Figure 7:**
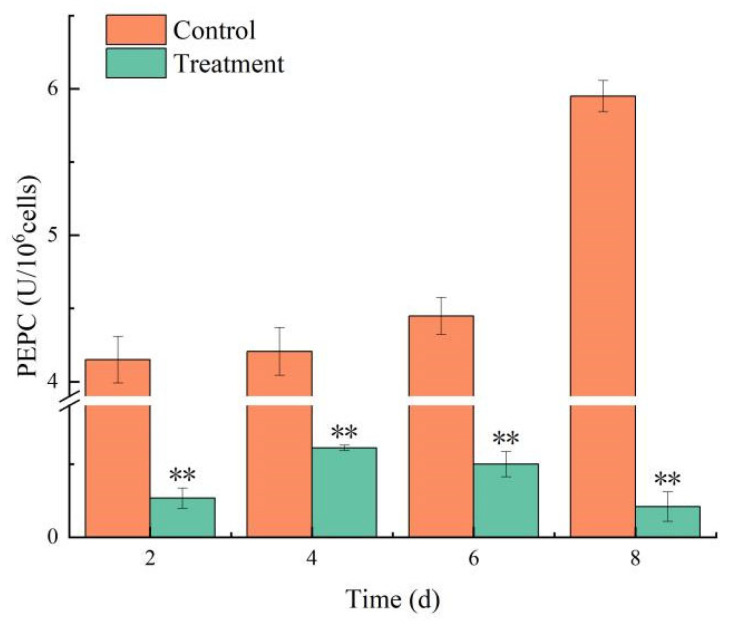
The effect of combined TBC and L-lysine on PEPC activity of *M. aeruginosa*. Note: ** represent statistically significant differences with respect to the values of control cultures at *p* < 0.01 level.

Under the influence of combined TBC and L-lysine, the PEPC activity in the control continued to rise, sustaining a level above 4.0 U/10^6^ cells. Conversely, PEPC activity in the treatment group plummeted to 0.2 U/10^6^ on day 8, corresponding to an inhibition rate of 96.48% (Figure 7). The combined TBC and L-lysine inhibited the physiological activities of the photosynthetic system in *M. aeruginosa*.

### 3.5. Effects of Combined TBC and L-Lysine on the Antioxidant Defense Response of M. aeruginosa

**Figure 8:**
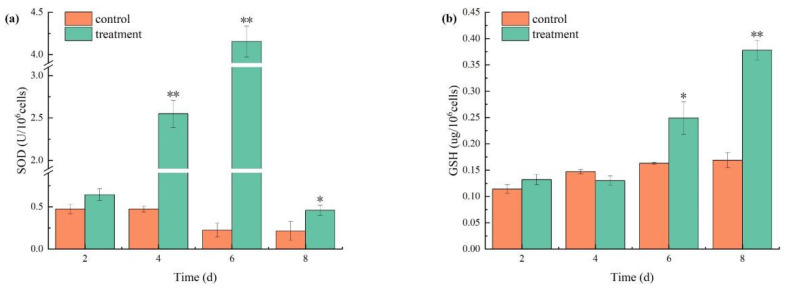
The effects of combined TBC and L-lysine on SOD (**a**) and GSH (**b**) activities of *M. aeruginosa*. Note: * and ** represent statistically significant differences with respect to the values of control cultures at *p* < 0.05 and at *p* < 0.01 levels, respectively.

SOD and GSH activities increased significantly in the cells in the treatment group. Initially, the SOD activity of *M. aeruginosa* exposed to combined TBC and L-lysine increased before subsequently decreasing (Figure 8a). SOD activity peaked at 4.15 U/10^6^ cells on day 6 before declining to 0.46 U/10^6^ cells, yet it remained above the control levels (Figure 8a). Additionally, the GSH activity of *M. aeruginosa* demonstrated a progressive increase over time (Figure 8b), with the treatment group exhibiting GSH activities that were 1.52 times those of the control on day 6 and 2.42 times those of the control on day 8.

### 3.6. Effects on Cell Membrane Permeability

**Figure 9:**
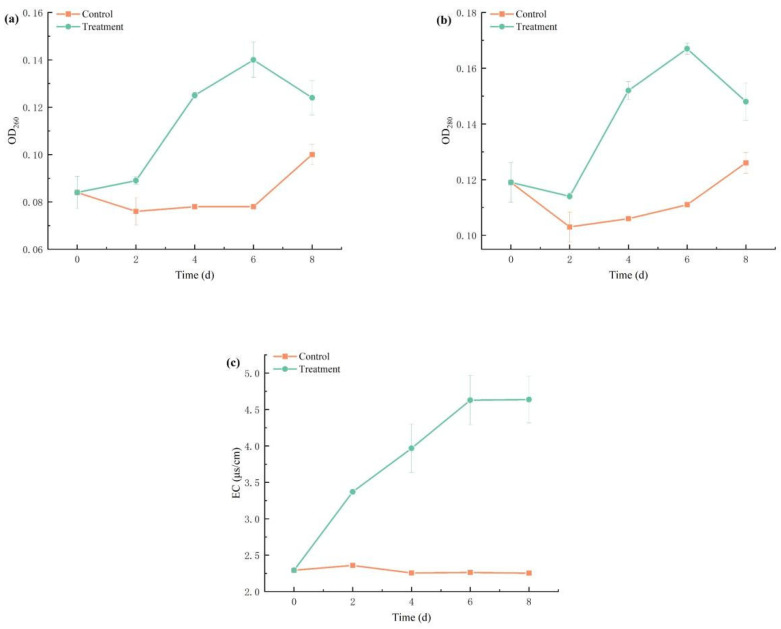
Effects of combined TBC and L-lysine on nucleic acid (**a**) content, protein (**b**), and conductivity (**c**) of *M. aeruginosa*.

Figure 9 presents dynamic alterations in nucleic acid content (OD_260_), protein leakage (OD_280_), and extracellular conductivity (EC) in *M. aeruginosa* cell membranes under combinatorial algaecide exposure. In the control group, OD_260_ remained stable, fluctuating between 0.07 and 0.08 from day 2 to day 6. In contrast, the treatment group demonstrated a gradual increase in OD_260_ from 0.09 to 0.14, followed by a decrease to 0.12 after day 6. A similar trend was noted for OD_280_, suggesting alterations in cell membrane permeability.

### 3.7. Production and Degradation of MCs

**Figure 10:**
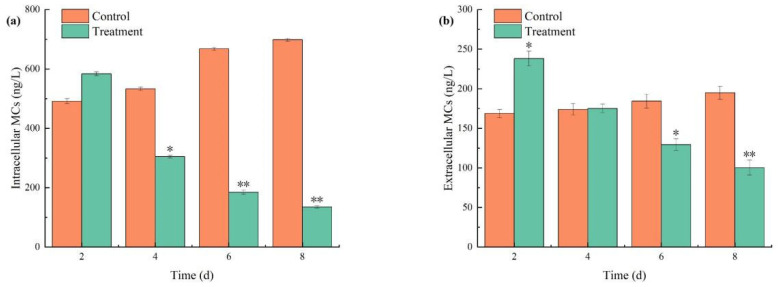
The effects of combined TBC and L-lysine on intracellular (**a**) and extracellular (**b**) MCs of *M. aeruginosa*. Nore: * and ** represent statistically significant differences with respect to the values of control cultures at *p* < 0.05 and at *p* < 0.01 levels, respectively.

Investigating the impact of the algicidal process on MCs release is critical, as shown in Figure 10. Compared to the control group, both intracellular and extracellular MCs initially increased before significantly declining under the combined algaecide treatment, with concentrations on day 2 surpassing those of the control group. As algal cell growth was markedly inhibited, the levels of MCs decreased. After 2 days of exposure, the concentrations of both intracellular and extracellular MCs continued to decline, reaching 130.30 ng/L and 97.95 ng/L by day 8, respectively. In contrast, the concentrations of MCs in the control group continued to rise, which was attributed to the increasing algal density over time.

## 4. Discussion

Our findings provide novel insights into the combined inhibitory mechanisms of TBC and L-lysine on *M. aeruginosa*; our findings differ from those of previous studies as they demonstrate not only growth inhibition but also significant suppression of microcystin production.

### 4.1. Combined Inhibition of M. aeruginosa Growth by TBC and L-Lysine

TBC and L-lysine can inhibit the growth of *M. aeruginosa* at low concentrations. In our study, treatment with 0.04 mg/L TBC resulted in a 77.61% inhibition rate of *M. aeruginosa*, which is consistent with previous findings that reported up to 77.2% inhibition at 0.04 mg/L [23]. Many researchers have investigated its inhibitory effects on harmful algae. Studies confirm L-lysine significantly reduces *M. aeruginosa* biomass while modulating key physiological processes, including photosynthesis and antioxidant defense mechanisms [24,25,26]. The experimental results demonstrate that L-lysine exhibits inhibitory effects on *M. aeruginosa* at low concentrations.

During Phase I of the combined inhibition experiments, the 0.03 mg/L TBC + 1 mg/L L-lysine treatment achieved significantly greater biomass reduction (*p* < 0.05) than the 0.03 mg/L TBC + 3 mg/L L-lysine treatment. Interestingly, the algal inhibition rate decreased with increasing concentrations of L-lysine. In Phase II, where different combination ratios were examined, the inhibitory effect did not consistently increase with higher concentrations of the combined substances. The optimal inhibition effect was specifically achieved with the combination of 0.04 mg/L TBC and 1 mg/L L-lysine. This observation aligns with recent studies that report varying inhibition effects on algal growth resulting from different allelochemical combinations at various ratios [27]. This phenomenon likely stems from divergent molecular targets among allelochemicals, resulting in characteristic nonlinear dose–response curves in combination treatments [28]. The reduced efficacy with certain combinations may be attributed to antagonistic interactions between the compounds. For instance, compared to EC_50_-equivalent concentration ratios, the combined application of caffeic acid and nonanoic acid at identical concentration ratios demonstrated an enhanced inhibitory efficacy against algal growth [29].

Recent studies have shown that combinations of allelochemicals can enhance the inhibition of harmful algae (Table 1) [30]. Specifically, ternary mixtures containing nonanoic acid, N-phenyl-1-naphthylamine, and caffeic acid demonstrate potent *M. aeruginosa* inhibition [29]. Experimental results showed that the combined application of 0.04 mg/L TBC and 1 mg/L L-lysine induced a continuous decline in *M. aeruginosa* cell density from day 0 to day 8, achieving an inhibition rate of 94.14% after 96 h. In comparison, TBC alone resulted in a 77.61% inhibition rate, while L-lysine alone led to only a 15.06% reduction. This combination exhibited substantially superior algal inhibition compared to either allelochemical applied separately. These results clearly indicate an interactive effect when the compounds are combined. Compared to previous studies, the optimized combination of TBC and L-lysine not only demonstrates enhanced algicidal efficacy but also offers reduced cost and broader practical application prospects.

In viable cells with intact membranes, fluorescein diacetate (FDA) is hydrolyzed intracellularly to produce fluorescein, which emits bright green fluorescence when excited with blue light. Moreover, the fluorescence intensity is directly proportional to the metabolic activity of the cells. When cells undergo apoptosis or death and exhibit compromised membrane integrity, propidium iodide (PI) can enter the cell, bind to nucleic acids, and produce red fluorescence [32]. FDA/PI dual staining flow cytometry quantitatively demonstrated time-dependent viability loss in *M. aeruginosa* cultures treated with the TBC–L-lysine combination.

### 4.2. Combined TBC and L-Lysine Treatment Impaired the Photosynthetic System of M. aeruginosa

Chlorophyll fluorescence characteristics provide valuable information regarding the electron transfer efficiency and function of photosystem II (PSII) in algae [33]. The combined TBC and L-lysine treatment significantly impacted the photosynthetic system of *M. aeruginosa*, as evidenced by reductions in chlorophyll fluorescence parameters including *F_v_*/*F_m_*, YII, α, and rETR_max_. These reductions suggest that the electron-donating side of the PSII reaction center represents a primary molecular target of the combinatorial algaecidal mechanism [34]. After 4 days of TBC treatment, *F_v_*/*F_m_*, rETR, and φP0 decreased sharply, indicating that the photosystem of *M. aeruginosa* was adversely affected, with a marked reduction in chlorophyll fluorescence parameters [23]. In contrast, 1.4 mg/L L-lysine monotherapy caused transient ΦPSII inhibition during the first 3 days, followed by partial recovery, indicating the incomplete suppression of the photosynthetic system [5]. Therefore, it can be inferred that TBC plays a dominant role in the combined treatment’s effect on photosynthesis [16]. Overall, the combined TBC and L-lysine treatment weakens the photosynthetic capacity of *M. aeruginosa* by significantly reducing its light capture ability and light energy utilization efficiency.

Chl-a, an important component of the photosynthetic system, absorbs light energy to sustain photosynthesis and participates in energy conversion [35]. Soluble protein content, a recognized biomass indicator, correlates directly with algal cell viability. Studies have demonstrated that L-lysine inhibits *M. aeruginosa* by reducing Chl-a content, with corresponding declines observed in soluble protein levels [36]. Under the combined treatment of TBC and L-lysine, both Chl-a and soluble protein contents exhibited a decrease over time. This decline likely results from disrupted pigment biosynthesis, leading to reduced photosynthetic efficiency, impaired light energy absorption/conversion, and eventual photosynthetic inhibition [37].

The combined TBC and L-lysine significantly reduced the contents of PC, APC, and PE, three light-capturing proteins located on phycobilisomes. Similarly, the combined inhibitory effect of luteolin and kaempferol on *M. aeruginosa* induced a dose-dependent suppression of phycobiliproteins (PC, APC, and PE) during the later phase of the experiment [12]. PC, APC, and PE primarily function as light-harvesting pigments that channel absorbed energy to chlorophyll, thereby facilitating algal photosynthesis [38]. The combined TBC and L-lysine treatment reduced the Chl-a content and impaired the photosystem of *M. aeruginosa*. Consequently, the algae produced substantial quantities of phycobiliproteins as a stress response to adapt to harsh environmental conditions. These findings illustrate that combined TBC and L-lysine can inhibit the photosynthesis of *M. aeruginosa* and damage chlorophyll by disrupting the synthesis of light-harvesting proteins in algal cells.

Phosphoenolpyruvate carboxylase (PEPC) is a pivotal enzyme in the photosynthetic pathway, playing a critical role in photosynthesis and also participating in amino acid metabolism [39]. In algal cells, an increase in PEPC activity can enhance the function of antioxidant enzymes, which can protect the algal cells from damage. Our study demonstrated that combined TBC and L-lysine treatment significantly reduced PEPC activity, which is consistent with previous reports that allelochemicals markedly inhibit PEPC levels in *M. aeruginosa* [40].

### 4.3. Oxidative Stress Response of M. aeruginosa to Combined TBC and L-Lysine

The redox imbalance and oxidative damage induced by allelochemicals can be detrimental to algae [41]. To counteract oxidative stress, algal cells have developed a highly efficient antioxidant system that includes enzymes such as superoxide dismutase (SOD) and antioxidants like glutathione (GSH) [42]. SOD functions as a primary antioxidant enzyme by catalyzing the dismutation of superoxide radicals into hydrogen peroxide during cellular metabolism. GSH constitutes an essential component of the antioxidant defense system, protecting cells from environmental oxidative stress. Furthermore, reduced GSH is responsible for scavenging reactive oxygen species (ROS) to ameliorate stress responses [43]. The dynamic equilibrium between oxidant production and antioxidant removal is crucial for maintaining cellular redox balance. Disruption of this equilibrium leads to oxidative stress, characterized by macromolecular damage and functional impairments [44].

The combined TBC and L-lysine disrupt the antioxidant defense system of *M. aeruginosa* by impairing intracellular redox homeostasis, which consequently affects the activities of SOD and GSH. Elevated ROS levels trigger compensatory upregulation of antioxidant enzymes to neutralize excessive superoxide radicals [45]. SOD catalyzes the dismutation of superoxide radicals (O_2_^−^) into hydrogen peroxide (H_2_O_2_), thereby reducing overall ROS levels and protecting cells from oxidative damage [46]. The observed increase in GSH activity likely represents a compensatory response to heightened oxidative stress, which may also stimulate the upregulation of other antioxidant enzymes [47]. Consequently, the increased SOD activity in combinatorial treatments may partially result from GSH gene overexpression. GSH scavenges ROS and alkyl peroxides, thereby protecting thiol-containing proteins and enzymes in the cell membrane from oxidative denaturation and maintaining cellular redox balance [48]. When treated with 0.04 mg/L TBC alone, *M. aeruginosa* showed significantly increased SOD and CAT content, along with marked upregulation of SOD and GSH genes, indicating an attack on the algal antioxidant defense system [23]. Similarly, L-lysine exposure triggered oxidative stress in *M. aeruginosa*, evidenced by elevated SOD activity [36]. Under combined treatment, a significant increase in SOD and GSH content was also observed, inducing oxidative stress and triggering cellular defense responses in *M. aeruginosa*.

### 4.4. Combined TBC and L-Lysine Altered Membrane Permeability in M. aeruginosa

Under normal conditions, cell membranes maintain intracellular homeostasis by regulating the exchange of substances. Increased membrane permeability facilitates leakage of intracellular contents, including small ions (K^+^, PO_4_^3−^) and macromolecules (DNA/RNA), into the extracellular environment. Initially, small ions like potassium and phosphate leak out, leading to an increase in EC, followed by larger molecules including DNA and RNA. Consequently, the leakage and release of electrolytes, nucleic acids, and proteins indicate irreversible damage to cell membranes. Under the combined TBC and L-lysine, the increase in EC in the algal suspension may be attributed to elevated cell membrane permeability under stress, resulting in the leakage of intracellular substances and a subsequent rise in conductivity [49]. The leakage of intracellular substances indicates cell membrane permeability related to cell membrane damage [50]. Under the combined TBC and lysine, damage to algal cell membranes occurs, leading to increased levels of extracellular proteins and nucleic acids (OD_280_ and OD_260_) in the algal suspension [51]. This phenomenon aligns with previous reports that indicate phenolic compounds cause irreversible damage to cell membranes, resulting in significant increases in extracellular protein and nucleic acid concentrations as well as electrical conductivity [52].

### 4.5. TBC and L-Lysine Co-Treatment Modulates Microcystin Synthesis in M. aeruginosa

During its growth–senescence cycle, *M. aeruginosa* synthesizes diverse secondary metabolites, including MCs—potent hepatotoxins with global prevalence in eutrophic waters [53]. Although *M. aeruginosa* typically upregulates MCs production as a protective response to external damage, extended exposure appears to overwhelm this adaptive mechanism [54,55]. The substantial accumulation of MCs within algal cells, when released into the water, can lead to cell rupture and subsequent death [56]. In this study, despite the algal inhibition process resulting in cell death, the concentration of extracellular MCs did not increase correspondingly; instead, a decreasing trend was observed. This reduction can be attributed to the inhibition of intracellular MCs synthesis, which causes cellular damage without a significant release of MCs. Additionally, Chl-a from *M. aeruginosa* and extracellular humic substances may have served as photosensitizers. Under the incubator’s light conditions, these compounds likely promoted MCs photodegradation, leading to partial MCs removal [57,58]. This suggests that the combined treatment of TBC and L-lysine is safe and environmentally friendly.

## 5. Conclusions

In this study, both TBC and L-lysine exhibited significant inhibitory effects on *M. aeruginosa*. At the combined concentration of 0.04 mg/L TBC and 1 mg/L L-lysine, the algal inhibition efficacy significantly exceeded the arithmetic sum of individual inhibitory effects from each allelochemical alone. Comprehensive analyses—including assessments of membrane integrity, growth performance, photosynthetic efficiency, oxidative stress responses, and microcystin (MC) dynamics—provided deeper insight into the underlying mechanisms of this combined treatment. Mechanistically, the combination regimen disrupted membrane integrity and suppressed photosynthesis, potentially perturbing metabolic pathways and signal transduction, while simultaneously upregulating antioxidant enzymes (SOD, GSH) to trigger oxidative stress. Notably, restricted MC release (both intra- and extracellular) during algal lysis highlights the environmental safety profile and practical feasibility of this combinatorial strategy. These findings establish TBC and L-lysine as an effective combination for *M. aeruginosa* control, and further research on their stability, degradation characteristics, and ecological safety could provide additional insights for practical applications.

## Figures and Tables

**Table 1:** The combined effect of allelochemicals.

Algae-Inhibiting Substances	Concentration (mg/L)	Inhibition Effect	Days (d)	Cost (¥/m^3^)	References
Luteolin and kaempferol	3.5 + 6.5	85%	14	197.4	[12]
Artemisinin, nonanoic acid, malonic acid, and ethyl acetate	3.94 + 6.27 + 8.2 + 6.38	more than 80%	7	31	[31]
Nonanoic acid and N-Phenyl-1-naphtylamine	1.25 + 1.25	85.43%	7	255	[29]
TBC and L-lysine	0.04 + 1	93.17%	4	0.28	This research

## Data Availability

The original contributions presented in the study are included in the article, and further inquiries can be directed to the corresponding author.
